# Comparison of Artificial Intelligence Techniques to Evaluate Performance of a Classifier for Automatic Grading of Prostate Cancer From Digitized Histopathologic Images

**DOI:** 10.1001/jamanetworkopen.2019.0442

**Published:** 2019-03-08

**Authors:** Guy Nir, Davood Karimi, S. Larry Goldenberg, Ladan Fazli, Brian F. Skinnider, Peyman Tavassoli, Dmitry Turbin, Carlos F. Villamil, Gang Wang, Darby J. S. Thompson, Peter C. Black, Septimiu E. Salcudean

**Affiliations:** 1 Department of Electrical and Computer Engineering, University of British Columbia, Vancouver, British Columbia, Canada; 2 Department of Urologic Sciences, University of British Columbia, Vancouver, British Columbia, Canada; 3 Department of Pathology and Laboratory Medicine, Vancouver General Hospital, Vancouver, British Columbia, Canada; 4 British Columbia Cancer Agency, Vancouver, British Columbia, Canada; 5 Richmond Hospital, Vancouver Coastal Health, Richmond, British Columbia, Canada; 6 Emmes Canada, Burnaby, British Columbia, Canada; 7 Department of Statistics and Actuarial Science, Simon Fraser University, Burnaby, British Columbia, Canada

## Abstract

**Importance:**

Proper evaluation of the performance of artificial intelligence techniques in the analysis of digitized medical images is paramount for the adoption of such techniques by the medical community and regulatory agencies.

**Objectives:**

To compare several cross-validation (CV) approaches to evaluate the performance of a classifier for automatic grading of prostate cancer in digitized histopathologic images and compare the performance of the classifier when trained using data from 1 expert and multiple experts.

**Design, Setting, and Participants:**

This quality improvement study used tissue microarray data (333 cores) from 231 patients who underwent radical prostatectomy at the Vancouver General Hospital between June 27, 1997, and June 7, 2011. Digitized images of tissue cores were annotated by 6 pathologists for 4 classes (benign and Gleason grades 3, 4, and 5) between December 12, 2016, and October 5, 2017. Patches of 192 µm^2^ were extracted from these images. There was no overlap between patches. A deep learning classifier based on convolutional neural networks was trained to predict a class label from among the 4 classes (benign and Gleason grades 3, 4, and 5) for each image patch. The classification performance was evaluated in leave-patches-out CV, leave-cores-out CV, and leave-patients-out 20-fold CV. The analysis was performed between November 15, 2018, and January 1, 2019.

**Main Outcomes and Measures:**

The classifier performance was evaluated by its accuracy, sensitivity, and specificity in detection of cancer (benign vs cancer) and in low-grade vs high-grade differentiation (Gleason grade 3 vs grades 4-5). The statistical significance analysis was performed using the McNemar test. The agreement level between pathologists and the classifier was quantified using a quadratic-weighted κ statistic.

**Results:**

On 333 tissue microarray cores from 231 participants with prostate cancer (mean [SD] age, 63.2 [6.3] years), 20-fold leave-patches-out CV resulted in mean (SD) accuracy of 97.8% (1.2%), sensitivity of 98.5% (1.0%), and specificity of 97.5% (1.2%) for classifying benign patches vs cancerous patches. By contrast, 20-fold leave-patients-out CV resulted in mean (SD) accuracy of 85.8% (4.3%), sensitivity of 86.3% (4.1%), and specificity of 85.5% (7.2%). Similarly, 20-fold leave-cores-out CV resulted in mean (SD) accuracy of 86.7% (3.7%), sensitivity of 87.2% (4.0%), and specificity of 87.7% (5.5%). Results of McNemar tests showed that the leave-patches-out CV accuracy, sensitivity, and specificity were significantly higher than those for both leave-patients-out CV and leave-cores-out CV. Similar results were observed for classifying low-grade cancer vs high-grade cancer. When trained on a single expert, the overall agreement in grading between pathologists and the classifier ranged from 0.38 to 0.58; when trained using the majority vote among all experts, it was 0.60.

**Conclusions and Relevance:**

Results of this study suggest that in prostate cancer classification from histopathologic images, patch-wise CV and single-expert training and evaluation may lead to a biased estimation of classifier’s performance. To allow reproducibility and facilitate comparison between automatic classification methods, studies in the field should evaluate their performance using patient-based CV and multiexpert data. Some of these conclusions may be generalizable to other histopathologic applications and to other applications of machine learning in medicine.

## Key Points

**Question:**

What is an appropriate approach to evaluate artificial intelligence classification methods in medical imaging such as histopathologic images of prostate cancer?

**Findings:**

In this quality improvement study, a classifier for automatic grading of prostate cancer in 333 digitized histopathologic images from 231 patients was found to significantly overestimate the prediction accuracy when using a patch-based cross-validation compared with when using a patient-based cross-validation. The classifier had less agreement with pathologists when it was trained on data from a single expert, rather than data from several experts.

**Meaning:**

This study suggests that patch-based training and evaluation of machine learning models may be flawed and should be avoided, and multiexpert data should be used to obtain a more realistic evaluation of model performance.

## Teaser

This quality improvement study compares several artificial intelligence (AI) cross-validation approaches to evaluate the performance of a classifier for automatic grading of prostate cancer in digitized histopathologic images, both when trained using data from 1 expert and when trained using data from multiple experts.

## Introduction

In the last decade, the literature on medical imaging in general, and on digital pathology in particular, has seen a dramatic increase in articles involving artificial intelligence and machine learning for automatic image analysis and classification,^1,2^ as part of the development of computer-aided diagnosis systems to increase accuracy, reproducibility, and efficient throughput. This trend has been enabled by an increase in computational power, improvement of image processing and machine learning algorithms, and the availability of more comprehensive data sets for training and evaluation.

Typical computer-aided diagnosis systems consist of a training phase and an inference phase. During training, a set of labeled instances (ie, instances with known inputs and outputs) is used to learn or determine the optimal values of the model parameters. The set of instances used in the training phase is referred to as the training set. If the output is a continuous number, such as blood pressure, the model is called a *regressor*. If, on the other hand, the output is a small number of discrete categories, such as benign and cancerous samples, the model is called a *classifier*. This work is focused on classifiers as we target the problem of classifying histopathologic images into several classes such as benign, low-grade cancer, and high-grade cancer.

A well-known pitfall when determining the model parameters from training data is overfitting, whereby the parameters are tuned such that the model performs very well on the training data but underperforms on other data. Therefore, the performance of the model should be evaluated on a separate set of instances, known as the *test set*. To avoid the overfitting problem and obtain an unbiased evaluation of the model, the data in the test set should be independent and separate from the data in the training set.

A common practice, known as *k-fold cross-validation* (CV),^3^ is to divide all of the available data into k partitions, or “folds,” of approximately equal sizes. Then, the model is trained k times from scratch. Each time, one of the k folds is held out to be used as the test data, and the remaining k − 1 folds are used as the training data. This approach allows one to make full use of the data by evaluating the model on all instances in an unbiased manner.

As opposed to other modalities, such as magnetic resonance imaging, in which entire images can be used as input, classification of digitized histopathologic images presents a challenge in processing because of their extremely large size, in the order of 0.1 to 10 gigapixels. To overcome this challenge, each whole slide image is divided into a grid of smaller image “patches,” which are typically square and only a few hundred pixels in length. Consequently, many instances are generated from the same slide and patient.

Prostate cancer is a heterogeneous disease that is manifested in a variety of very different histopathologic patterns across patients. Because of its heterogeneity, grading of prostate cancer has a well-known high degree of interobserver variability^4,5,6^ that leads to uncertainty in image labeling. Therefore, training and evaluation of a classification algorithm against 1 expert may involve an inappropriate ground truth, which would yield a classifier that underperforms when evaluated against other experts. The Figure illustrates the interobserver variability on a tissue core from our data set.

**Figure.**
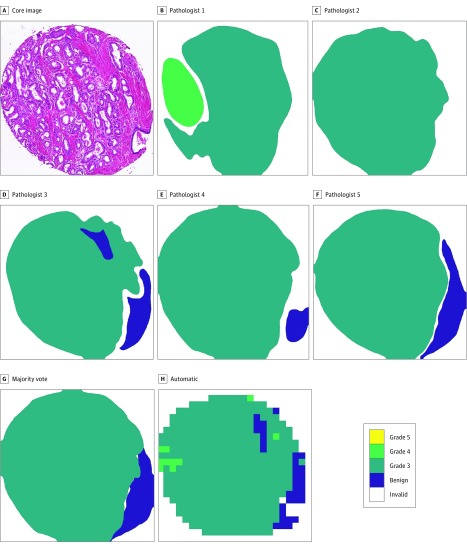
Interobserver Variability in Grading of Prostate Cancer in Hematoxylin-Eosin–Stained Tissue Scanned at ×40 Magnification The contours represent the detailed annotations of each pathologist on an example tissue core. The majority-vote label based on the annotations is overlaid on the image, as well as the automatic classification result of each patch (a small rectangular subimage) based on one of the cross-validation experiments. Gleason grade 3 indicates low-grade cancer and grades 4 and 5 indicate high-grade cancer.

We report our experience in automatic grading of prostate cancer in histopathologic images based on annotations by multiple experts, and examine different approaches to evaluation of these images. The motivation for this work arose from our interest to compare our results^7^ with those of other reported studies. In this process, we found the evaluation approaches to be inconsistent across literature reports. In particular, we found that many studies have used data from a single expert to train and evaluate their models,^8,9,10,11^ a practice that clearly ignores the extensive evidence of high interobserver variability. More important, we found that some studies had followed a patch-based CV, in which patches from all patients are included in the training and test sets.^10,11^ A patch-based CV is fundamentally different from a patient-based CV; since the final test of the usefulness of automatic classification is whether a new patient can be correctly assessed based on the experience from other patients, it seems that patient-based CV is the only correct way of validating machine learning models for medical applications. Whereas in a patient-based CV, data from certain patients are held out during model training, in patch-based CV there is no such guarantee. Even if there is no overlapping between adjacent patches extracted from images, a patch-based CV presents a quite different problem because patches extracted from a patient are likely to include much information that is unique to that patient.

Table 1 summarizes the evaluation approach and results of a sample of studies on prostate histopathologic characteristics.^7,8,9,10,11,12^ Only a few studies have used a patient-based evaluation on multiexpert data.^7,12^ Our goal is to demonstrate the importance of the evaluation method of artificial intelligence techniques when applied in this field, to assist clinicians in interpreting the widely divergent results reported in the literature. We aim to guide researchers in this field to improve their experimental design and performance evaluation and to avoid biased evaluations that can lead to inconsistent and erroneous conclusions.

**Table 1.** Selected Previous Work

Source	Classification Type	Data	Multiple Experts	Validation Type	Results
Monaco et al,^8^ 2010	Cancer vs benign	20 Patients and 40 slides	No	Slide-based cross-validation	Sensitivity, 0.87 and specificity, 0.90
Doyle et al,^9^ 2012	Cancer vs benign	58 Patients and 100 slides	No	Slide-based and patient-based	Image-based accuracies, 0.69, 0.7, and 0.69; patient-based accuracies, 0.74, 0.66, and 0.57
Gorelick et al,^10^ 2013	Cancer vs benign; high-grade vs low-grade	15 Patients, 50 slides, and 991 patches	No	Patch-based cross-validation	90% Cancer vs benign, and 85% high-grade vs low-grade
Nguyen et al,^11^ 2014	Benign, Gleason grade 3, and Gleason grade 4	29 Patients and 317 patches	No	Patch-based 10-fold cross-validation	87.3% Accuracy for Gleason grade 3 vs 4
Arvaniti et al,^12^ 2018	Gleason grades 3, 4, and 5	641 + 245 Patients and unspecified number of patches	Yes (training on 1, evaluated on 2)	Patient-based (1 partitioning)	58% Recall on test set
Nir et al,^7^ 2018	Gleason grades 3, 4, and 5	231 Patients, 333 cores, and approximately 16 000 patches	Yes (6 for training and testing)	Patient-based cross-validation (repeated partitioning)	Benign vs cancer: accuracy, 90.2%, sensitivity, 91.3%, and specificity, 84.0%; Gleason grade 3 vs grade 4-5: accuracy, 76.6%, sensitivity, 75.9%, and specificity, 77.9%

## Methods

Our data comprised 7 tissue microarray slides that contained tissue cores sampled from radical prostatectomy specimens. Sections of the blocks were stained in hematoxylin-eosin and digitized as virtual slides at ×40 magnification using a SCN400 Slide Scanner (Leica Microsystems). This study was approved by the Clinical Research Ethics Board of the University of British Columbia. The patient data were deidentified. Patients consented to the use of their data in research projects, including our own. This study followed the Standards for Quality Improvement Reporting Excellence (SQUIRE) reporting guidline.

A subset of 333 tissue cores were sampled from 231 patients who underwent radical prostatectomy at the Vancouver General Hospital between June 27, 1997, and June 7, 2011. The cores were independently graded in detail (4 classes: benign and cancer Gleason grade 3, grade 4, and grade 5) between December 12, 2016, and October 5, 2017 by 6 pathologists (L.F., B.F.S., P.T., D.T., C.F.V., and G.W.) who included a research-based genitourinary pathologist, 4 clinical genitourinary pathologists, and a clinical general pathologist, ranging from midcareer to veteran, with 1 to 27 years of experience (median, 16 years) in prostate cancer grading. Four of the pathologists annotated all 333 cores. The other 2 pathologists each annotated 191 and 92 cores.

We divided each core into a grid of rectangular patches of 192 µm.^2^ These patches were used to train a convolutional neural network^13^ in different experiments. The convolutional neural network model used in this work is based on the MobileNet architecture that is well suited for applications with small training data sets.^14^ For the CV experiments we used the majority-vote label of each patch among the pathologists as the true class, while for the cross-experts experiments we used the label of each pathologist.

Our CV experiments were performed as follows:

1. A 20-fold leave-patients-out CV. In this experiment, we first randomly shuffled the cores from all 231 patients, then divided them into 20 folds. Each fold included 11 or 12 patients. We trained our convolutional neural network model 20 times, each time using the data from 19 folds as the training data and the data from the held-out fold as the test data.
2. A 20-fold leave-cores-out CV. We divided the 333 cores into 20 folds, with each fold containing 16 or 17 cores. Therefore, some cores in the training and test folds can belong to the same patient.
3. A 20-fold leave-patches-out CV. In this experiment, all patches extracted from all cores were randomly assigned to 20 folds. We trained 20 models, with each model using 19 folds as the training set, and tested the model on the held-out fold.
4. A 2-fold leave-patches-out CV. All patches were randomly divided into 2 folds. Hence, in this setting we trained 2 models. Each model is trained on 50% of the data and tested on the remaining 50%.

To evaluate the model performance, we computed the accuracy, sensitivity, and specificity for cancer detection (benign vs cancer) and cancer grading of low-grade (Gleason grade 3) vs high-grade (Gleason grade 4 and 5) cancer.

We performed another set of experiments to study the difference between single-expert vs multiple-expert data. This set of experiments followed a 20-fold leave-patients-out CV, as described above. We trained the convolutional neural network classifier using the labels of a single pathologist in each experiment, and then computed the agreement level of the model with the labels of every pathologist on the held-out patients. For each fold, we also trained the classifier using the labels of the majority vote among the pathologists, and repeated the same evaluation with the pathologists.

As a common metric to evaluate agreement, we used the (quadratic) weighted κ statistics (Cohen κ coefficient).^15^ The coefficient ranges from –1 to 1. Nonpositive values indicate an accidental agreement; positive values in the interval of 0.1 to 0.2 reflect slight agreement, positive values in the interval of 0.21 to 0.4 reflect fair agreement, positive values in the interval of 0.41 to 0.6 reflect moderate agreement, positive values in the interval of 0.61 to 0.8 reflect substantial agreement, and positive values in the interval of 0.81 to 1.0 reflect (near) perfect agreement. The quadratic-weighted κ assigns larger weights to larger errors between annotators while downplaying smaller errors. For example, a grading difference of Gleason grade 4 and Gleason grade 5 between 2 annotators will receive a much smaller weight than a difference of benign and Gleason grade 5, which is clinically much more significant.

### Statistical Analysis

Analysis was performed between November 15, 2018, and January 1, 2019. For a statistical comparison of different CV methods, we used the McNemar test.^16^ In each of our CV experiments, each patch was tested exactly once. For comparing each pair of CV methods (eg, 20-fold leave-patches-out vs 20-fold leave-patients-out), we formed a contingency table that showed the count of patches on which each of the 2 methods made correct and incorrect predictions. The null hypothesis in the McNemar test is that the probability that method 1 makes a correct prediction and method 2 makes an incorrect prediction is equal to the probability that method 1 makes an incorrect prediction and method 2 makes a correct prediction. Using this test, we computed the *P* value for the observed data under the null hypothesis. All *P* values were from 2-sided tests and results were deemed statistically significant at *P* < .001.

## Results

On data from 231 patients with prostate cancer with a mean (SD) age of 63.2 (6.3) years (a total of 333 tissue microarray cores), 20-fold leave-patches-out CV for classification of patches into cancer vs benign classes resulted in mean (SD) accuracy of 97.8% (1.2%), sensitivity of 98.5% (1.0%), and specificity of 97.5% (1.2%). The 20-fold leave-patients-out CV for the same classification task resulted in mean (SD) accuracy of 85.8% (4.3%), sensitivity of 86.3% (4.1%), and specificity of 85.5% (7.2%). Similarly, 20-fold leave-cores-out CV resulted in mean (SD) accuracy of 86.7% (3.7%), sensitivity of 87.2% (4.0%), and specificity of 87.7% (5.5%). Results of McNemar tests showed that the leave-patches-out CV accuracy, sensitivity, and specificity were significantly higher than those for both leave-patients-out CV and leave-cores-out CV. Even though the difference between patient-based CV and core-based CV was not large (we expect this because there are only 2 cores per patient), results of the McNemar test showed that this difference was also statistically significant. The 2-fold leave-patches out CV resulted in mean (SD) accuracy of 96.8% (0.0%), sensitivity of 96.8% (0.0%), and specificity of 97.2% (0.0%). Similar results were observed for classifying low-grade cancer vs high-grade cancer. A summary of all results for CV experiments are available in Table 2. Table 3 shows the results of the McNemar test for comparison accuracies of different methods in classifying benign vs cancerous patches.

**Table 2.** Results of the Cross-Validation Experiments

Cross-Validation Method	Classification	Accuracy, Mean (SD), %	Sensitivity, Mean (SD), %	Specificity, Mean (SD), %
20-Fold leave-patients-out	Benign vs cancer	85.8 (4.3)	86.3 (4.1)	85.5 (7.2)
	Low-grade vs high-grade	81.2 (3.7)	82.4 (5.0)	82.0 (8.1)
20-Fold leave-cores-out	Benign vs cancer	86.7 (3.7)	87.2 (4.0)	87.7 (5.5)
	Low-grade vs high-grade	83.4 (4.5)	86.2 (6.4)	84.2 (4.9)
20-Fold leave-patches-out	Benign vs cancer	97.8 (1.2)	98.5 (1.0)	97.5 (1.2)
	Low-grade vs high-grade	92.2 (4.5)	93.8 (5.8)	90.8 (6.0)
2-Fold leave-patches-out	Benign vs cancer	96.8 (0.0)	96.8 (0.0)	97.2 (0.0)
	Low-grade vs high-grade	87.0 (0.0)	84.1 (0.0)	94.1 (0.0)

**Table 3.** Results of the McNemar Test for Comparison of Different Cross-Validation Methods in Terms of Their Accuracy in Classifying Image Patches as Benign or Cancerous

Cross-Validation Method	*P* Value			
	20-Fold Leave-Patients-Out	20-Fold Leave-Cores-Out	20-Fold Leave-Patches-Out	2-Fold Leave-Patches-Out
20-Fold leave-patients-out	NA	NA	NA	NA
20-Fold leave-cores-out	<.001	NA	NA	NA
20-Fold leave-patches-out	<.001	<.001	NA	NA
2-Fold leave-patches-out	<.001	<.001	<.001	NA

Abbreviation: NA, not applicable.

When trained on a single expert, the overall agreement in grading between pathologists and the classifier ranged from 0.38 to 0.58; when trained using the majority vote among all experts, it was 0.60. The full results of the cross-expert experiments are summarized in Table 4 and show that training and evaluating the classifier on a single pathologist yields, in most cases, higher agreement levels (the diagonal elements in the table) than evaluating it on other pathologists (off-diagonal elements).

**Table 4.** Results of the Cross-Expert Experiment^a^

Source of Label Used as Ground Truth for Training	Agreement of the Automatic Classifier						
	With Pathologist 1	With Pathologist 2	With Pathologist 3	With Pathologist 4	With Pathologist 5	Pathologist 6	Overall
Pathologist 1	0.48	0.39	0.40	0.36	0.37	0.31	0.38
Pathologist 2	0.35	0.61	0.45	0.44	0.50	0.44	0.48
Pathologist 3	0.40	0.58	0.64	0.61	0.58	0.52	0.58
Pathologist 4	0.33	0.57	0.60	0.63	0.57	0.57	0.57
Pathologist 5	0.40	0.57	0.59	0.55	0.61	0.50	0.58
Pathologist 6	0.38	0.52	0.51	0.53	0.46	0.47	0.50
Majority vote of all pathologists	0.46	0.63	0.65	0.65	0.60	0.59	0.60

^a^ The values represent the (quadratic) weighted κ agreement between the corresponding automatic classifier and pathologists.

## Discussion

The results of our CV experiments showed marked and statistically significant differences in the accuracy, sensitivity, and specificity for different CV methods. Leave-patches-out CV led to very high accuracy, sensitivity, and specificity estimates. As we anticipated, comparing these values with those obtained with leave-patients-out CV and leave-cores-out CV showed a significant difference. The accuracy, sensitivity, and specificity for the leave-patches-out CV were significantly higher. Even the results of 2-fold leave-patches-out CV were significantly better than the results of 20-fold leave-patients-out CV and 20-fold leave-cores-out CV, even though the number of patches available for training in 2-fold leave-patches-out CV is approximately half that available for 20-fold leave-patients-out CV and 20-fold leave-cores-out CV.

Thus, using patchwise evaluation may lead to highly overestimated performance and misinterpretation of the results, especially by a nontechnical audience. This large difference is because patch-based and patient-based evaluation represent 2 quite different problems. Patient-based CV is the only correct way of evaluating machine learning methods because it takes into consideration the interpatient variation in the phenomena that are being modeled, which in this study are the histologic patterns in the prostate biopsy tissue. Patch-based CV, on the other hand, completely ignores such variation by including patches from all patients in both training and test sets. Hence, a patch-based CV is a flawed experimental design because the observed prediction accuracy will not be generalizable to unseen patients, which is the true goal of a machine learning model in medical applications.

Our experiments with single-expert data and multiple-expert data demonstrate the importance of evaluating the classification performance across multiple experts rather than a single expert. The results suggest that studies in the literature that used a single pathologist are likely to have overestimated their classification performance.

To our knowledge, this study is the first to systematically compare patch-based CV vs patient-based CV and to study single-expert data vs multiple-expert data for the important task of prostate cancer prediction and grading in digital histopathologic images using machine learning models. Our results show that both these factors are important to the generalizability and interpretability of the results. Hence, our results indicate that for these models to be effective in classifying new patients, they should be trained using patient-based CV on multiexpert data.

As can be seen from the example studies listed in Table 1, in general, studies that have used patch-based evaluation have reported higher accuracy values, although a direct and unbiased comparison is not possible because of the differences in the size and nature of the data and the type of classification algorithms used. Our patient-based accuracy, sensitivity, and specificity values are within the range of those reported by recent studies.^7,12^ Our patch-based evaluation results are better than those seen in previous studies.^10,11^ This is likely because of the higher representation capacity of the deep learning classifier that we used.

One way to address this interobserver variability is to apply a multilabel classifier that takes into account the multiple annotations during the training process. The overall agreement levels of the classifier that was trained by the majority vote of the 6 pathologists was higher than those obtained using a single pathologist. In most cases, the agreement of the majority vote classifier with an individual pathologist was even higher than the agreement of a single pathologist–trained classifier with the very same pathologist it was trained on, which suggests that training using the multilabel approach improved the generalizability of the classifier.

Another approach to using multiple-expert data, rather than using the majority vote, is to assign weights to different labels based on prior or learned information regarding the quality of the annotations of each expert. A study on automatic grading of prostate cancer in digital pathology^7^ adapted a crowdsourcing algorithm^17^ that computes the sensitivity and specificity of each expert annotator for each class and estimates a ground truth on which a classifier can be trained.

### Limitations

The main limitation of this study is that it has been restricted to prostate cancer prediction and grading from tissue microarray histopathologic images. We think that our arguments regarding the flawed nature of patch-based training and validation and the need for patient-based analysis hold for other digital pathology applications and also for other applications of machine learning methods in medicine. We also expect that the benefits of using multiple-expert data observed in this study should extend to many other applications in medicine. However, each application may warrant a separate investigation to determine whether these factors have a statistically significant effect.

## Conclusions

In this work, we demonstrated that some of the studies on prostate cancer classification from histopathologic images that have been published in recent years have followed flawed experimental designs. Specifically, we showed that patch-based training and evaluation could lead to significant overestimation of a model’s predictive accuracy. We also showed that training on data provided by a single expert can lead to biased results that have poor generalizability compared with a model trained on data from multiple experts.

Our results show that patient-based training and evaluation is the only acceptable method for developing machine learning models in this application. Furthermore, to improve reproducibility and generalizability of the results and to facilitate comparison between different works, annotation data by multiple experts should be used to develop and evaluate these models. We expect that our conclusions may apply to other fields in digital histopathology and medical image analysis in general. However, independent studies are warranted to determine the significance of these factors in each application. The method that we proposed in this study can be followed to establish the significance of these factors in other application areas of machine learning and artificial intelligence in medicine.
